# An Evaluation of MTA Cements as Coronal Barrier

**Published:** 2006-10-01

**Authors:** Zahed Mohammadi, Abbasali Khademi

**Affiliations:** 1 *Department of Endodontics, Dental School, Shahid Sadoughi University of Medical Sciences, Yazd, Iran*; 2 *Department of Endodontics, Dental School, Isfahan University of Medical Sciences, Isfahan, Iran*

**Keywords:** Bacterial Leakage, Coronal Barrier, MTA, Resin Modified Glass Ionomer

## Abstract

**INTRODUCTION:** Coronal leakage seems to play an important role in the failure of endodontic treatment. A double seal over root canal filling has been suggested as a means of improving the coronal seal. Several restorative materials have been used in an attempt to produce a coronal barrier. The purpose of this study was to assess gray-coloured mineral trioxide aggregate (GMTA), white-coloured mineral trioxide aggregate MTA (WMTA), and Principle (a resin-modified glass ionomer) as coronal barriers to bacterial leakage.

**MATERIALS AND METHODS:** Fifty-one human anterior teeth were cleaned and obturated with gutta-percha and sealer. In group 1, teeth received a 3 mm barrier of GMTA. In groups 2 and 3, samples received WMTA and Principle, respectively. Obturated teeth without barrier were used as positive control and obturated teeth covered with epoxy resin were used as negative control. A leakage model utilizing Enterococcus faecalis used for the evaluation. Leakage was recorded when turbidity was observed.

**RESULTS:** All controls behaved as expected. Three samples in group 1, three samples in group 2, and four samples leaked in group 3. There was no statistically significant difference in leakage between GMTA and WMTA or between GMTA and Principle.

**CONCLUSION:** It seems that GMTA, WMTA and Principle can be recommended as a coronal barrier for up to 90 days.

## INTRODUCTION

Root canal treatment failures have been attributed to many causative factors. Coronal leakage, among these factors, is of very great importance (1). Leakage studies have demonstrated that the loss of the coronal seal provides a route for bacterial recontamination of endodontically treated teeth (2-6). A lack of the coronal seal may lead to endodontic failure (7). Delay in placement of a permanent restoration, fracture of the coronal restoration and/or tooth, inadequate thickness of the temporary restoration, and post space preparation with inadequate remaining apical filling are potential means of coronal recontamination of obturated root canals (8).

Swanson and Madison (8) found that after obturation of the root canals, coronal microleakage in the presence of saliva was inevitable up to 85% of the time. Several other studies confirmed the importance of adequate coronal seal (2-8). A double seal over root canal filling (intra-orifice barrier) has been suggested as a means of improving the coronal seal. 

Several restorative materials have been used in an attempt to produce a coronal barrier with varying results. One of these materials, mineral trioxide aggregate (MTA) has been evaluated for a wide variety of applications (9). Gray version of MTA (GMTA) was introduced to endodontics in 1993 (10).

MTA is a powder that consists of fine hydrophilic particles that sets in the presence of moisture. The main reason that makes MTA attractive is its ability to resist leakage as well as superior marginal adaptation (9). Newer formulation of MTA is white in color (WMTA). The only chemical difference between the GMTA and the WMTA is the reduced iron content in WMTA. Additionally, the particle size of the WMTA is smaller to enhance handling and placement characteristics (11-12).

Despite the wide range of potential applications, few studies have been conducted to evaluate MTA as a coronal barrier (13-15). The purpose of this study was to compare GMTA, WMTA and a RMGI, Principle as coronal barriers.

## MATERIALS AND METHODS

Fifty-one extracted human central incisors were used in this study. The teeth were examined and radiographed and those presenting radicular calcifications or anatomical abnormalities were excluded. The teeth were stored in 0.2% thymol solution immediately after extraction and kept moist before and during the experiment. An access opening was prepared using a high-speed hand piece and a #2 round bur with a constant water spray. A #10 file was used to establish working length and to maintain patency. Working length was established by measuring the length at which a #10 file was first visible at the apical foramen and subtracting 1 mm. Canals were cleaned and shaped using K3 rotary files in a crown down fashion. Two milliliters of 2.6% sodium hypochlorite was used during instrumentation for canal irrigation between each file size. Canals were then dried with paper points and were obturated using gutta-percha and AH-26 sealer (Dentsply, Germany) with a lateral condensation technique. The gutta-percha level was reduced using a hot instrument to a depth of 3mm from the cementoenamel junction (CEJ). The level of gutta-percha reduction was verified radiographically. Teeth were randomly divided into three experimental groups of 15 teeth each and two control groups of 3 teeth each. Coronal orifice of each experimental specimen was covered to a depth of 3 mm with one of the test materials. The materials used were GMTA (Group1) WMTA (Group2), and Principle (Group3). All materials were mixed according to the manufacturer’s instruction. The positive control group consisted of three teeth obturated in the same manner as experimental teeth without a coronal barrier. The negative control group consisted of three obturated teeth without a coronal barrier, but with crowns and roots covered completely with epoxy resin. Glass tubes equipped with microcaps were used to suspend the prepared teeth in Brain Heart infusion (BHI) broth. A hole was made at the centre of each cap and tooth was placed into the hole to the CEJ. The gap between the tooth and the hole was filled with sticky wax.

The completed apparatus was then sterilized by autoclaving. A 24h broth culture of E. faecalis was placed into the pulp chamber of the tooth suspended in sterile BHI broth to the level sufficient for covering the apical 3mm of the root tip. Tubes were incubated at 37°C until the BHI broth became turbid, indicating bacterial growth. Fresh 24h cultures of E. faecalis were added every two days throughout the study. Turbidity of the broth was monitored daily for a total period of 90 days. The data were analyzed using the Fisher exact test at 95% level of confidence.

## RESULTS

In group 1, leakage was observed in three samples. In groups 2 and 3 leakage was observed in three and four samples, respectively. All positive controls demonstrated leakage. None of the negative controls leaked. Leakage did not occur until day 55 with Principle, day 57 with GMTA and day 59 with WMTA. There was no statistically significant difference in leakage between GMTA and WMTA or between GMTA and Principle.

## DISCUSSION

The results of the present study indicated that MTA_when placed coronally in 3mm thickness over gutta-percha_ significantly reduced bacterial penetration. Barrieshi-Nusair and Hammad (1), in an in vitro study, compared the sealing ability of MTA and glass ionomer (GI) as intraorifice barriers using a dye leakage model. Their results showed that MTA group leaked significantly less than GI group.

However, in the present study a bacterial leakage model was used. It appears that bacterial leakage tests are more reliable for testing different root canal filling materials and techniques. Furthermore, the accuracy of dye leakage results may depend on the chemical properties of the test materials and the dye used (17).

MTA and resin modified GIs (RMGIs) seem to be promising materials to prevent coronal leakage.Although MTA has been used in a variety of applications, there are few published studies on the use of MTA to improve the coronal seal (13-15). An in vivo study showed that after 10 months, there were no demonstrable differences between periapical inflammation in dogs’ teeth with conventional root fillings and those coronally augmented by MTA (13). Cummings and Torabinejad (14) compared MTA with IRM and zinc phosphate cement as a coronal barrier for internal bleaching. MTA demonstrated superior performance. However, they did not test the efficacy of MTA as a coronal barrier against microbial leakage. In another in vitro study, Tselnik et al. (15) reported that there was no statistically significant difference between GMTA, WMTA and Fuji II LC in coronal leakage for up to 90 days, which confirms the findings of the present study.

Principle is a RMGI which is not only capable of free- radical polymerization, but its acidic functional groups also react with the glass filler and tooth structure to promote adhesion.

In the present study, Principle demonstrated an acceptable coronal seal for up to 90 days. Mavec et al. (16) reported that the use of 3 mm of vitrebond (RMGI) as a coronal barrier in post-prepared teeth with significantly extended time of leakage, which is similar to the results of the present study.Wells et al. (17) compared Principle to C & B Metabond as a coronal barrier. They found that Principle leaked significantly more than C & B Metabond at 1 h, but the its seal improved at 4- weeks. In another study, it was reported that Fuji II LC similar to GMTA and WMTA provided an acceptable coronal seal for to 90 days (15) which confirms the results of the present study.

The superior performance of RMGI may be explained by water sorption of the material which will result in setting expansion, and consequently a better seal.

Another property of RMGI is the release of fluoride, which may decrease coronal leakage by increasing its antimicrobial activity.

## CONCLUSION

Based on the results of the present study, GMTA, WMTA, and principle were effective in improving coronal seal in endodontically treated teeth.
